# Nanoparticles as catalysts of agricultural revolution: enhancing crop tolerance to abiotic stress: a review

**DOI:** 10.3389/fpls.2024.1510482

**Published:** 2025-01-17

**Authors:** Yahan Cao, Khalid Turk, Nabila Bibi, Abdul Ghafoor, Nazeer Ahmed, Muhammad Azmat, Roshaan Ahmed, Muhammad Imran Ghani, Muhammad Abass Ahanger

**Affiliations:** ^1^ Key Laboratory of Plant Resource Conservation and Germplasm Innovation in Mountainous Region (Ministry of Education), College of Life Sciences, Guizhou University, Guiyang, Guizhou, China; ^2^ Center for Water and Environmental Studies, College of Agricultural and Food Sciences, King Faisal University, Al-Ahsa, Saudi Arabia; ^3^ Department of Botany, The Islamia University of Bahawalpur, Bahawalpur, Pakistan; ^4^ Laboratory of Green Pesticide and Agricultural Bioengineering, Ministry of Education, Guizhou University, Guiyang, Guizhou, China; ^5^ Department of Biology, College of Science, University of Lahore, Lahore, Pakistan; ^6^ Department of Plant Pathology, Faculty of Agriculture and Environment, The Islamia University of Bahawalpur, Bahawalpur, Pakistan; ^7^ College of Agriculture, Guizhou University/College of Life Sciences, Guiyang, China; ^8^ Key Laboratory for Tropical Plant Improvement and Sustainable Use, Xishuangbanna Tropical 20 Botanical Garden, Chinese Academy of Sciences, Menglun, China

**Keywords:** climate change, nanotechnology, abiotic stress, nanoparticles, sustainable agriculture, plant stress mitigation

## Abstract

Ensuring global food security and achieving sustainable agricultural productivity remains one of the foremost challenges of the contemporary era. The increasing impacts of climate change and environmental stressors like drought, salinity, and heavy metal (HM) toxicity threaten crop productivity worldwide. Addressing these challenges demands the development of innovative technologies that can increase food production, reduce environmental impacts, and bolster the resilience of agroecosystems against climate variation. Nanotechnology, particularly the application of nanoparticles (NPs), represents an innovative approach to strengthen crop resilience and enhance the sustainability of agriculture. NPs have special physicochemical properties, including a high surface-area-to-volume ratio and the ability to penetrate plant tissues, which enhances nutrient uptake, stress resistance, and photosynthetic efficiency. This review paper explores how abiotic stressors impact crops and the role of NPs in bolstering crop resistance to these challenges. The main emphasis is on the potential of NPs potential to boost plant stress tolerance by triggering the plant defense mechanisms, improving growth under stress, and increasing agricultural yield. NPs have demonstrated potential in addressing key agricultural challenges, such as nutrient leaching, declining soil fertility, and reduced crop yield due to poor water management. However, applying NPs must consider regulatory and environmental concerns, including soil accumulation, toxicity to non-target organisms, and consumer perceptions of NP-enhanced products. To mitigate land and water impacts, NPs should be integrated with precision agriculture technologies, allowing targeted application of nano-fertilizers and nano-pesticides. Although further research is necessary to assess their advantages and address concerns, NPs present a promising and cost-effective approach for enhancing food security in the future.

## Introduction

### Climate change and agriculture

Abrupt environmental changes are intensifying their negative impacts on the negative impacts on crop physiology, growth, development, and productivity. Environmental stressors such as drought, salt, temperature stress, and HM toxicity have become more frequent and severe, leading to a substantial decline in global agricultural output. This situation is further compounded by the growing global population, projected to exceed 9 billion by 2050, intensifying the demand for food systems to sustainably increase production.

Traditional chemical-based farming practices have been proven proven insufficient in meeting these demands. The extensive dependence on agrochemicals and pesticides has significant adverse effects, including soil degradation, a decline in biodiversity, and environmental pollution (Tripathi et al., 2020; Brunelle et al., 2024). For example, excessive use of nitrogen-based fertilizers, induces soil acidification, which reduces nutrient availability and disrupts the soil’s microbial ecosystem, ultimately lowering soil productivity. Pesticide use, particularly with organophosphates and carbamates, has reduced populations of beneficial soil microorganisms, weakening soil structure and fertility. In addition, traditional irrigation practices have exacerbated issues like water scarcity and soil salinity (Mohanavelu et al., 2021; Tarolli et al., 2024). These challenges highlight the urgent need for sustainable agricultural technologies to enhance crop resilience to environmental stressors and improve yield.

### Impacts of abiotic stress on crop production

Agriculture is essential for maintaining global food security and supporting the livelihoods of a large segment of the global population. Therefore, protecting this vital sector from environmental stressors, particularly those intensified by climate change, is crucial. Abiotic stresses, such as drought, salinity, and HM toxicity, are responsible for 20-50% of annual global crop yield losses. Under adverse conditions, plants experience impaired physiological functions, including reduced photosynthesis, nutrient absorption, and water uptake, which can slow growth and, in extreme cases, lead to complete crop failure, especially in sensitive species like maize (Li et al., 2021b).

Abiotic stress encompasses external factors that disrupt normal plant processes and cause physiological and biochemical changes that impact agricultural yield. These stressors, such as severe climatic conditions, including drought, salinity, and temperature fluctuations, disrupt normal plant processes and lead to physiological and biochemical changes, ultimately impacting agricultural yield (Ghani et al., 2022; Kareem et al., 2022; Hassan et al., 2023). Drought stress, for instance, significantly decreases nitrogen uptake efficiency, diminishing the effectiveness of fertilization, while salinity stress imposes osmotic pressure on plants, increasing their vulnerability to other abiotic factors. These combined environmental stressors can significantly reduce the adaptive capacity of ecosystems to respond to fluctuating environmental conditions (Han et al., 2022; Anwar et al., 2023).

Climate change poses significant threats to agriculture in the modern world, impacting crop physiology, growth, development, and productivity.

### Role of nanotechnology in agriculture

**Figure 1:**
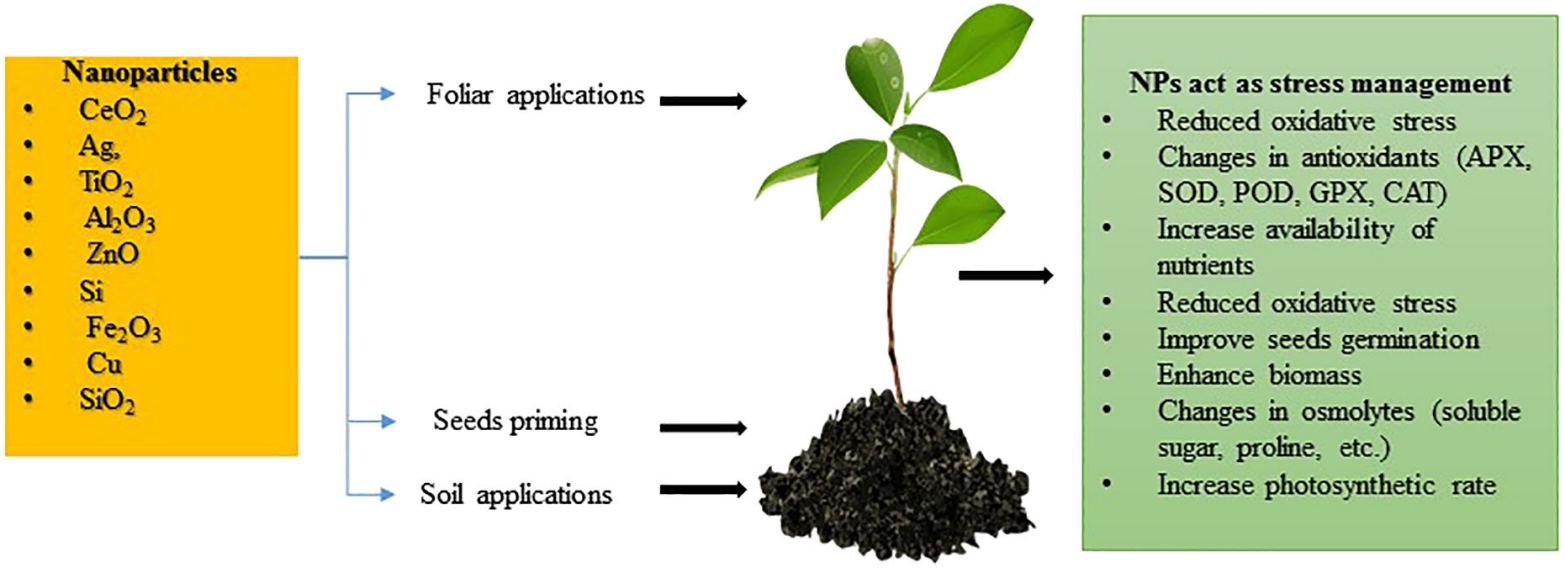
Effect of different NPs and their different applications on seed germination, performance and physiological parameters and photosynthetic rate.

Nanotechnology, particularly the use of NPs, has emerged as a key player with significant potential to enhance crop resilience and productivity. NPs exhibit special physicochemical properties, including a large surface-area-to-volume ratio and the ability to cross biological membranes, facilitating targeted nutrient delivery and stress resistance (Singh et al., 2024). NPs can enhance nutrient uptake, improve stress resistance, and increase photosynthetic efficiency (**Figure 1**), resulting in better growth and yield even in challenging conditions (Singh et al., 2024).

Advancement in agriculture, particularly via the adoption of nanotechnology, is essential in the modern world. NPs possess exceptional chemical and physical properties that enable them to penetrate cellular membranes and interact effectively with biological systems. In agriculture, nanoscale products, such as nano-pesticides, nano-fungicides, nano-herbicides, and nano-fertilizers, offer significant benefits. They can improve plant growth and reduce the environmental footprint of conventional agricultural chemicals (Wahab et al., 2024). These nanoparticles are effective even at low concentrations, and can be delivered through various methods, including seed treatment, foliar spraying, and hydroponic delivery (Nile et al., 2022; Mawale et al., 2024). Unlike traditional fertilizers that often leach into water bodies and cause pollution, nanoparticles provide controlled, efficient nutrient release, ensuring plants receive the necessary elements over time (Easwaran et al., 2024; Haydar et al., 2024). For instance, they can improve the delivery of vital nutrients like nitrogen (N), phosphorus (P), and potassium (K), reducing environmental runoff and eutrophication (Sheikhalipour et al., 2021; Jakhar et al., 2022). Nanotechnology also offers potential in mitigating specific abiotic stresses, such as drought and salinity, by enhancing water-use efficiency and osmotic adjustment in plants. Although the application of NPs in agriculture is still in its developmental stages, it holds promise for increasing crop resilience against various stressors (Su et al., 2019; Haydar et al., 2024) (**Table 1**). An overview of studies investigating the impact of various NPs on different plant components is provided.

**Table 1:** Impacts of nanoparticles on different growth levels of plants.

Nanoparticles	Plants	Plants response	References
CeO_2_	*Sorghum*	Improve seed yield and carbon assimilation rates in leaves	(Djanaguiraman et al. 2018)
	*Glycine max*	Improve plants growth	(Cao et al. 2018)
	*Triticum aestivum*	Improve biomass, grain yield, and shoot growth	(Kusiak et al. 2022)
Ag	*Eruca sativa*	Enhanced length of roots	(Bhattacharjee et al. 2022)
	*Oryza sativa*	Improved root growth, biomass	(Yang et al. 2018)
	*Lolium multifolium*	Improve plant growth and enzymatic activity	(Faizan et al., 2023)
	*Vigna sinensis*	Increased growth and biomass through encouraging root growing and bacterial diversity in the soil	(Pallavi et al. 2016)
	*Triticum aestivum*	Better growth and heat stress resistance	(Iqbal et al. 2019)
TiO_2_	*Cicer arietinum*	Change the status of redox reaction, improve photosynthetic content	(Verma et al. 2022)
	*Spinacia oleracea*	Enhanced chlorophyll content, plant growth	(Wani et al. 2023)
	*Oryza sativa*	Increased antioxidant capacity and decreased Cd translocation to reduce Cd toxicity and enhance growth	(Rizwan et al. 2019)
	*Citrullus lanatus*	Increased the roots activity, MDA level	(Rui et al. 2016)
Al_2_O_3_	*Glycine max*	Improve plant growth, enzyme activity	(Hossain et al. 2016)
	*Raphanus sativus*	Increased root length, root shoot biomass	(Ahmad et al., 2023)
	*Solanum lycopersicum*	Effectively in control Fungus-induced tomato root rot	(Shenashen et al. 2017)
	*Zea mays*	Root length increased, reduced attack of pathogens	(Aparato & Suh 2022)
ZnO	*Cyamopsis tetragonoloba*	Impressive rise in chlorophyll, leaf protein, and alkaline phosphate content	(Ragab et al., 2022)
	*Arachis hypogaea*	Improved seed germination rate, chlorophyll content	(Al Jabri et al. 2022)
	*Prunus domestica fruits*	B.cinerea's suppressed symptoms of grey mold and soil-borne diseases	(Malandrakis et al. 2019)
	*Cicer arientum*	The plant's weight increased, reducing ROS production	(Srivastav et al. 2023)

### Challenges and considerations for nanotechnology use in agriculture

NPs are gaining attention in agriculture as a highly effective strategy for reducing abiotic stresses and serving as nano-fertilizers. They can stimulate plant growth, enhance nutrient uptake, and strengthen resilience to environmental stressors.

Despite their potential, the widespread adoption of nanoparticles in agriculture faces challenges. Concerns remain about the long-term effects of NPs on plant systems, soil microorganisms, and human health. For instance, while nanoparticles can enhance plant health, their impact on non-target organisms and overall ecosystem balance is not fully understood. Additionally, the cost-effectiveness of nanoparticle production is a critical factor that influences their feasibility for large-scale agricultural use. Addressing these concerns is vital for the responsible integration of NPs in agriculture. Ensuring the safety, environmental compatibility, and economic viability of nanotechnology will be essential to its sustainable implementation. This review aims to explore how nanoparticles can address agricultural challenges and identify environmentally friendly solutions. The main objectives are: (1) to analyze the adverse effects of abiotic stresses, such as drought, salinity, HM, and temperature changes, on crop yield and assess whether nanoparticles can mitigate these stressors and enhance yield; (2) to discuss the role of nanomaterials, including stable synthetic variants, in agriculture and evaluate their safety for humans and the environment; (3) to examine the cost-effectiveness of producing and utilizing nanoparticles in agricultural activities and determine their efficacy; and (4) to propose strategies for the safe incorporation of nanotechnology into agricultural practices and measures to optimize nanoparticle use.

### Mechanisms of nanoparticles in enhancing crop resilience

#### Nanoparticles physicochemical properties

NPs, due to their distinct characteristics, are considered effective in alleviating abiotic stress in plants. Their large surface area relative to volume increases reactivity, facilitating more efficient interactions with plant cells (El-Saadony et al., 2022). This property allows NPs to dissolve readily and penetrate plant cell membranes, thereby enabling the targeted delivery of nutrients and stress-mitigating agents (Yadav et al., 2023). Once NPs penetrate plant tissues either through root uptake or foliar retention, they translocate throughout the plant using via both apoplastic and symplastic routes. This localization and movement within plant tissues contribute to increased resistance against various abiotic stress factors, ultimately stimulating plant growth under challenging conditions (Ali et al., 2021b). By reaching intracellular targets, NPs can directly influence metabolic processes directly, improving nutrient uptake and stress resistance. This capability is particularly beneficial in enhancing photosynthetic efficiency and promoting plant growth under adverse environmental conditions (Singh et al., 2021b).

#### Defense mechanism

Plants have developed various defense mechanisms to cope with environmental stressors, utilizing both biochemical and physiological pathways to minimize damage (Haile et al., 2020; Hassan et al., 2023). While ongoing research continues to unravel these complex protective processes, certain mechanisms have been identified as essential for plant resilience. NPs are emerging as a promising tool for enhancing these defenses, as they help to strengthen plant defense by boosting the antioxidant activity, promoting, osmolyte accumulation, and activating genes associated with stress tolerance (Ali et al., 2019; Ahmed et al., 2021).

One of the primary ways NPs aid plant defenses is by enhancing antioxidant systems, which are crucial for neutralizing reactive oxygen species (ROS). Under conditions of environmental stress, such as drought, salinity, and UV exposure plants produce higher levels of ROS, which can damage cellular components like lipids, proteins, and DNA. NPs, particularly those containing titanium dioxide (TiO₂) and silicon (SiO₂), have shown the ability to alleviate oxidative stress by increasing the activity of key antioxidant enzymes, including superoxide dismutase (SOD) and catalase (CAT) (Ye et al., 2019). For example, TiO₂ NPs can function as catalysts in redox reactions, helping to detoxify ROS and protect plant tissues from UV-induced damage. Similarly, SiO₂ NPs have been found to enhance antioxidant defenses in wheat, helping to mitigate the damage caused by UV-B radiation. This enhancement of antioxidant activity helps maintain cellular integrity under stress, enabling plants to better endure challenging conditions.

In addition, to bolstering antioxidant defenses, NPs stimulate the accumulation of osmolytes, small organic compounds such as trehalose, proline, and glycine betaine, that play a crucial role in osmoregulation. These compounds help plants retain water, stabilize proteins, and protect cell membranes during periods of water deficit and high salinity conditions (Sarkar et al., 2024). Proline, for instance, acts as an osmoprotectant by maintaining cell turgor and facilitating cellular functions in low-water conditions. Similarly, glycine betaine and trehalose support membrane stability and protein structure under stress. By promoting osmolyte buildup, NPs help plants manage osmotic stress, improving their resilience in arid and saline environments.

NPs also appear to enhance stress tolerance at the genetic level by activating the expression of certain stress-related genes. Research has shown that NPs like silicon (SiO₂) and TiO₂ can trigger the transcription of genes involved in stress responses, including those that encode heat-shock proteins (HSPs) and late embryogenesis abundant (LEA) proteins (Zulfiqar and Ashraf, 2021a). These proteins play a critical role in protecting cellular structures under extreme stress conditions, further enhancing the plants ability to survive in harsh environments. By influencing these molecular pathways, NPs not only improve immediate stress resistance but may also contribute to longer-term adaptation to adverse conditions.

In general, the application of NPs in agriculture presents a potential approach for increasing crop resilience by bolstering plant defenses by using their distinct physicochemical characteristics and capacity to trigger the plant defense response. This approach improves nutrient delivery, stress resistance, and supports sustainable agricultural productivity in the face of increasing environmental challenges.

### UV radiation and oxidative stress in plants

Ultraviolet (UV) radiation is a significant abiotic stressor that induces oxidative stress in plants. Exposure to UV-B light leads to abnormal leaf structure and reduced photosynthetic efficiency, prompting plant cells to produce ROS. Additionally, UV-B exposure increases lipid peroxidation and the extracellular release of electrolytes through the generation of H_2_O_2_ and O_2_^-^ radicals. The elevated levels of ROS may result from the inhibition of SOD and APX activities due to UV-B (Takshak and Agrawal, 2014). TiO_2_ nanoparticles operate as catalysts in an oxidation-reduction process that produces superoxide anion radicals and hydroxide when exposed to light (Zou et al., 2013). Si NPs boost antioxidant activity in wheat, helping to reduce oxidative damage induced by UV-B exposure (MS et al., 2021). This can be inferred that Si NPs provide protection to plants by triggering the antioxidant defense system and mitigating the photosynthetic damage caused by ROS. Si NPs are more effective than bulk silicon in reducing UV-B stress in wheat seedlings (Ayub et al., 2022).

Multi-walled carbon nanotubes (MWCNTs) protect the model plant Arabidopsis thaliana by alleviating paraquat-induced oxidative stress. MWCNTs enhance photosynthetic efficiency and stimulate plant lateral root growth, and mitigate the bioavailability of toxic substances (Fan et al., 2018; Igiebor et al., 2023). NPs have demonstrated beneficial effects in mitigating oxidative stress in plants, but it is essential to carefully design approaches to minimize their possible toxicity and optimize their properties. Factors such as size, shape, and surface characteristics should be tailored to ensure the optimal performance of NPs while minimizing environmental risks (Chen et al., 2023).

### Optimizing nanoparticle properties for enhanced efficacy and reduced toxicity

NPs have shown the potential to mitigate oxidative stress in plants, improving their tolerance to environmental stressors (Thiruvengadam et al., 2024). However, careful design is crucial to minimize any risks associated with toxicity, ensuring NPs deliver their benefits without harmful side effects to plants or ecosystems. Therefore, safe and efficient production strategies should emphasize refining nanoparticle properties to maximize beneficial effects while minimizing adverse impacts (Iavicoli et al., 2017). In this regard, developing nanoparticles with optimized properties such as size, shape, and surface characteristics becomes essential for maximizing benefits while minimizing environmental risks. Smaller nanoparticles tend to be more efficiently absorbed and transported within plant tissues, and specific shapes, such as rod-like or tubular structures, can enhance interactions with plant cells (Zuverza-Mena et al., 2017; Wang et al., 2023). Additionally, surface modifications, such as applying biocompatible coatings, further improve nanoparticle stability, reduce aggregation, and lower toxicity by regulating how these particles interact with cell membranes (Mansouri et al., 2023). By carefully customizing these features, researchers can create nanoparticles that support plant health in a safer, more sustainable way, advancing eco-friendly applications of nanotechnology in agriculture (Yang et al., 2017a).

### Dose-dependent effects of nanoparticles on plant growth and toxicity

Previous research has demonstrated that the toxicity of NPs is highly dependent on their concentration, exposure time, and specific plant species (Ma et al., 2015; Cox et al., 2016; Khan et al., 2021). Certain NPs, such as TiO_2_, have demonstrated the ability to enhance plant development at low concentrations, while excessive accumulation at higher concentrations can lead to the overproduction of ROS, ultimately inhibiting growth. This dose-dependent response underscores the importance of carefully controlling NP exposure levels to achieve the desired beneficial effects while avoiding potential phytotoxicity (Yang et al., 2017b). TiO_2_ NPs were also demonstrated to increase SOD activity, with the influence increasing with NPs concentration. At 40 and 50 mg/mL concentrations, TiO_2_ NPs resulted in the lowest CAT and POD activity, whereas POD and amylase enzyme secretion were highest and lowest, respectively (Javed et al., 2022). The findings of these studies suggest that NPs are important for enhancing agricultural productivity, however, a full understanding of the right process and how NPs interact with plants at different levels is still evolving (Fincheira et al., 2020).

Jampílek and Kráľová (2022) investigation has shown that CuNPs exert an impact on the development of *Oryza sativa* (rice) and *Lactuca sativa* (lettuce). At low concentrations (0.8 to 798.9 mg/L), CuNPs promoted root growth. However, the phytotoxicity of this substance escalated with increasing concentration, leading to a reduction in the number of thylakoids and stomatal conductivity. This indicates that while low and medium doses are beneficial to plants, high doses can be toxic.

Various metal oxide nanoparticles, including those of copper, zinc, and silver, have been found to exhibit phytotoxic effects on plants. The magnitude of these hazards may vary across the crop, species, and cultivars (Xiong et al., 2017; Mahawar et al., 2024). Understanding the specific phytotoxic thresholds and mechanisms for each type of NP is crucial for developing safe and effective applications in agriculture (Tuga et al., 2023). CuNPs stressed *Oryza sativa*, resulting in a decrease in the rate of photosynthetic activity, the number of thylakoids in each granum, the rate of transpiration, and the conductivity of the stomatal cells (Aqeel et al., 2022).

ZnO NPs also exhibit phytotoxicity. Exposure to 100 and 1000 mg/L ZnO NPs in Salicornia persica resulted in a 50% reduction in shoot length, accompanied by increased levels of ROS production and lipid peroxidation compared to untreated plants. Similar effects on growth parameters were observed in *Cajanus cajan* seeds exposed to varying concentrations of ZnO NPs (Tortella et al., 2023). Additionally, the phytotoxicity of ZnO NPs is influenced by soil pH and plant species. Notably, acidic soils exhibited more pronounced toxic effects compared to alkaline soils, where the presence of Zn is restricted. The aforementioned observation suggests that environmental variables substantially influence the phytotoxic reaction to ZnO NPs (Fasake et al., 2021). It is crucial to emphasize that both soil pH and plant species significantly affect the phytotoxicity of ZnO NPs, as well as the damage caused by metal or metal oxide nanoparticles (Sturikova et al., 2018).

Cytotoxicity and phytotoxicity are also associated with silver nanoparticles (AgNPs). For instance, biogenic AgNPs synthesized using Aloe vera extract caused significant injury to Brassica seedlings in hydroponic systems. This stress resulted from oxidative effects that led to cell death and DNA damage. Although the toxicity of AgNPs was not as pronounced as that of silver nitrate, they still exhibited significant toxicity in certain concentrations (Feregrino-Pérez et al., 2023).

The surface characteristics of AgNPs significantly influence their phytotoxicity. NPs with a negative charge and Ag⁺ ions are particularly detrimental to plants (Sarkar et al., 2022). In contrast, titanium dioxide (TiO_2_NPs) are generally less phytotoxic than many other metal oxide nanoparticles, making them safer for plant applications (Irshad et al., 2021).

AgNPs with distinct properties can restrict the growth in model monocot and dicot plants to varying degrees. The synthesis of these AgNPs involved the use of trisodium citrate, tannic acid, and cysteamine hydrochloride, leading to the formation of nanoparticles with well-defined surface charges. Exposure to these silver nanoparticles adversely affected the roots and shoots of both monocot and dicot plants. Notably, negatively charged AgNPs and silver ions derived from silver nitrate (AgNO_3_) exhibited greater toxicity to plants (Cox et al., 2016).

Although TiO_2_ NPs have been assessed for their phytotoxic effects, they are generally less harmful to plants compared to metal or metal oxide NPs (Rastogi et al., 2017). The application of TiO_2_ NPs may pose less risk in terms of phytotoxicity, particularly if growth inhibition, alterations in root water transport, cell membrane damage, ROS generation, or chlorophyll synthesis inhibition are observed (Mir et al., 2021). Recent studies suggest that while TiO_2_ NPs can be phytotoxic, they may also exhibit hormetic effects, promoting shoot and root elongation as well as overall biomass growth at specific concentrations, such as 100 mg/L. However, these beneficial effects diminish at higher concentrations, particularly beyond 1000 mg/L (Maluin et al., 2021). However, there was a noticeable limitation at concentrations over 1000 mg/L. The use of NPs in plants has shown a number of favorable impacts. To avoid harm and unfavorable effects on non-target species, the dose must be calculated based on crop type, soil, and other considerations (Tortella et al., 2023).

### Long-term impacts of nanoparticles on soil ecosystem

While the integration of nanoparticles (NPs) into agricultural practices offers promising benefits for crop resilience and productivity, understanding their long-term effects on soil ecosystems remains crucial. Research has shown that NPs can persist in the environment and accumulate in soil, potentially altering the soil’s chemical and biological properties. The interactions between NPs and soil microorganisms, which play a pivotal role in maintaining soil health and nutrient cycles, are particularly concerning. Changes in microbial community structures can affect soil fertility, organic matter decomposition, and nutrient availability, potentially leading to disruptions in ecosystem services over time (Bakshi et al., 2015; Donia and Carbone, 2019).

The behavior of NPs in soil is influenced by several factors, including their size, surface charge, and coating, as well as soil pH and organic matter content. Studies indicate that certain metal and metal oxide nanoparticles, such as zinc oxide (ZnO) and TiO_2_, can exert phytotoxic effects that may vary with soil type and plant species (Ghori et al., 2019). Additionally, the formation of a protein corona around NPs can affect their absorption and movement through soil matrices, potentially impacting the bioavailability of essential nutrients and toxic metals (Huang et al., 2017).

Understanding the cumulative effects of NPs on soil ecosystems is vital for assessing their long-term sustainability. Potential outcomes include reduced microbial diversity, shifts in enzymatic activity, and altered soil-plant interactions, which could compromise agricultural productivity in the long run. These impacts necessitate further investigation through long-term field studies that assess NP behavior, transport, and transformation in soil environments under different climatic conditions.

### Management of abiotic stress from nanoparticles

Plants encounter numerous challenges from abiotic stressors, including drought, salinity, alkalinity, submersion, and deficiencies in minerals and metals. These environmental factors hinder plant growth and significantly reduce agricultural productivity (Ma et al., 2015; Li et al., 2021b; Kareem et al., 2022; Mahawar et al., 2024). Among these, salinity, drought, extreme temperatures, and heavy metals (HMs) are the predominant factors contributing to decreased agricultural productivity. Plants experience a range of abiotic stressors throughout their life cycle and have evolved sophisticated physiological processes to withstand these challenges (Ma et al., 2015; Ghani et al., 2022; Anwar et al., 2023). In response to these stressors, plants modulate gene expression to adapt and alleviate their impact, therefore facilitating their ability to handle unfavorable environmental conditions (Pathak et al., 2021).

Plants have to deal with several forms of abiotic stress throughout their life cycle and have developed various defense mechanisms to address them via various physiological pathways. Plants reduce and adapt to varied stressors by altering gene expressions. Experiments have demonstrated that nanoparticles help plants survive abiotic stresses by exerting a concentration-dependent result on plant growth. NPs enhance plants’ ability to withstand certain toxic metals, such as chromium (Cr), cadmium (Cd), iron (Fe), aluminum (Al), and manganese (Mn) (Faizan et al., 2023). 

**Figure 2:**
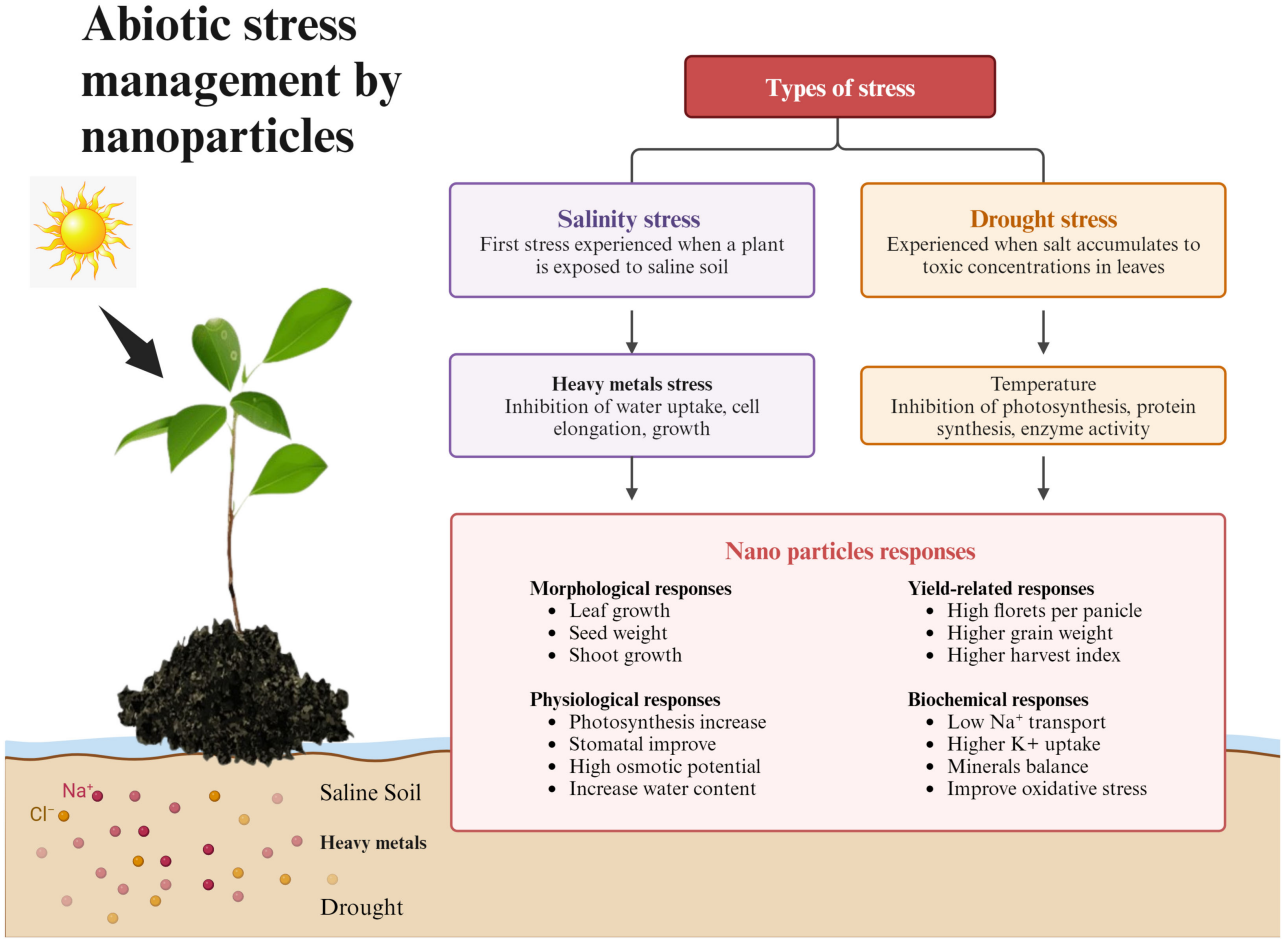
Flowchart demonstrating abiotic stress management by NPs and plants show response on different levels like morphological, yield related, physiological, and biochemical.

The absorption and movement of Cd, Mn, and Pb in plant tissues have also been shown to be decreased by the management of NPs. Furthermore, through increasing antioxidant activity, osmolyte accumulation, synthesis of free amino acids, and nutritional improvements, NPs help plants resist environmental challenges (Khalid et al., 2022). NPs are used to mitigate the harm caused by abiotic stress. Metal nanoparticles have several uses in plants. Si NPs encourage plant development and increase plant resilience to biotic stress (Yang et al., 2017b). The use of Cu, ZnO, and Se NPs as nano-fertilizers has shown great results. Additionally, nanoparticles have demonstrated the capacity to act as inducers of phytohormone production, controlling plant improvement and metabolism in response to abiotic stress (Zulfiqar and Ashraf, 2021b) **Figure 2**. illustrates abiotic stress management by modulating different morphological and physiological parameters.

### Drought stress

Drought is a significant challenge for agriculture, affecting farmers worldwide due to insufficient irrigation and reduced rainfall (Orimoloye et al., 2022). This natural phenomenon is complex and not limited to specific regions or timeframes, making it difficult to monitor and manage (Faiz et al., 2021). Drought stress is primarily caused by a decrease in precipitation, leading to prolonged dry periods. It manifests in four distinct forms: meteorological, hydrological, agricultural, and socio-economic (Haile et al., 2020). Dry weather conditions characterize meteorological drought, while hydrological drought is characterized by low water supply affecting surface and groundwater levels. Agricultural drought results from decreased soil moisture, leading to crop failures and impacting global food production. Socio-economic drought results from limited water supply, which affects economic activities (Swain et al., 2022).

**Figure 3:**
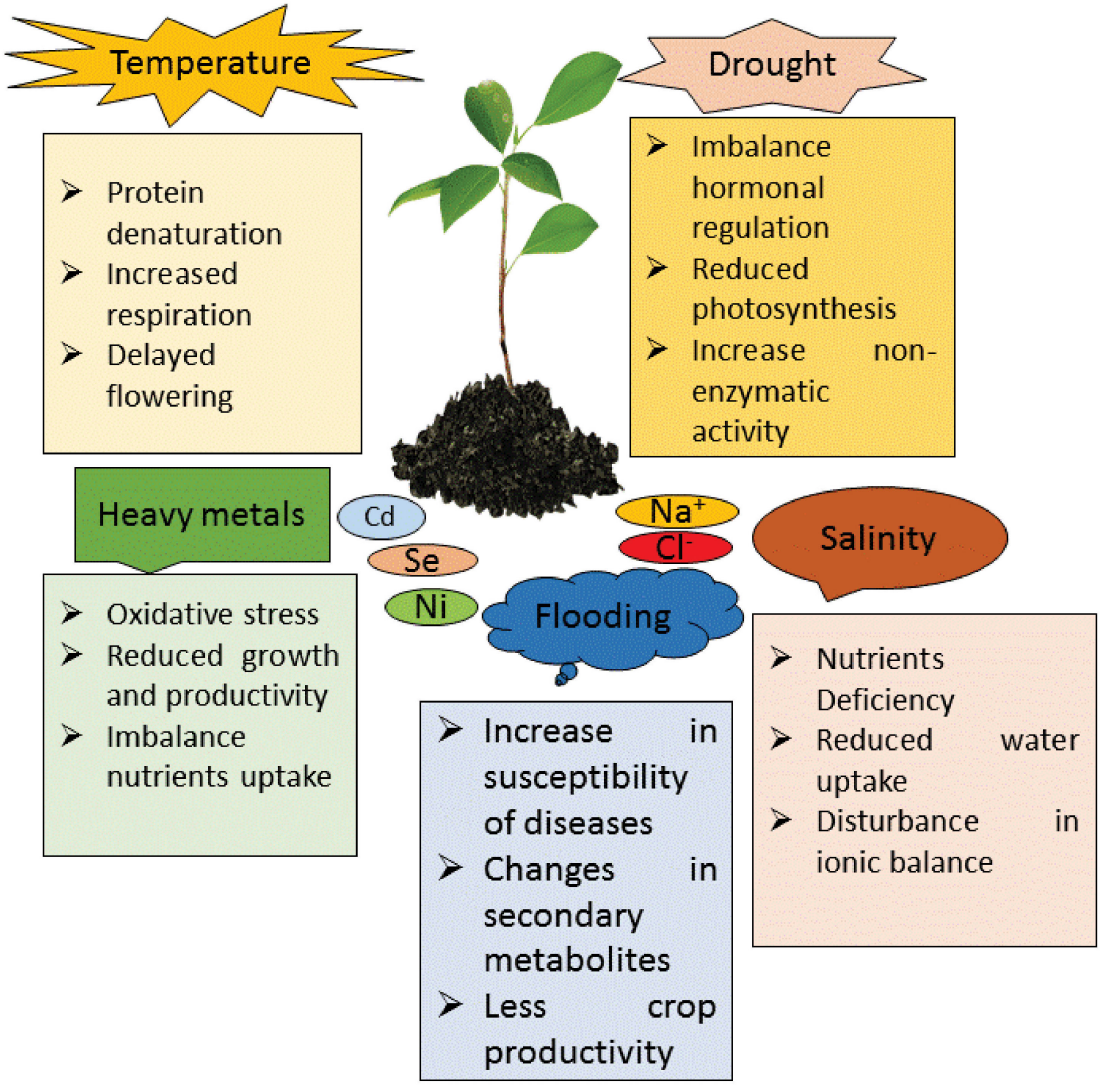
Impacts of drought stress and NPs on plants, showing morphological, physiological, and biochemical changes at different growth levels.

Plant physiological, biochemical, molecular, and genetic responses to drought and salinity are quite similar (Abdelaal et al., 2021; Ghani et al., 2022). Because water intake becomes constrained when soluble solute levels rise due to a drop in water potential, drought conditions start to occur more often in high-salinity locations (Ahluwalia et al., 2021). Plants respond to these stresses by generating phytohormones, adjusting osmotically, reducing transpiration, and altering growth, photosynthesis, carbon absorption, and leaf turgor (Hamid et al., 2021). **Figure 3** shows drought stress mitigation through different physiological and biochemical mechanisms.

NPs like Si, Ag, Zn, TiO_2_, and Fe enhance photosynthetic rates, reduce malondialdehyde levels, raise relative water content, and strengthen root and shoot systems, promoting plant growth and development (Chandrashekar et al., 2023) (**Table 2**). Summarize recent research on the role of NPs in alleviating drought stress. Although the precise methods by which nanoparticles provide drought resistance are not completely known, research indicates that they influence aquaporins—water channel proteins responsible for water transport and seed germination. This mechanism increases the availability of water and nutrients, enhancing germination rates even under dry situations (Zia et al., 2021).

**Table 2:** Role of nanoparticles in alleviating drought stress in plants.

NPs	Plants	Effect on plants	References
Chitosan	*Triticum aestivum*	Enhanced chlorophyll and relative water content	(Behboudi et al. 2019)
Si	*Triticum aestivum*	Decreased oxidative stress and increased chlorophyll concentration	(Khan et al. 2020)
Fe-CTs	*Mentha piperita*	Increases peppermint growth and yield, especially during drought conditions	(Giglou et al. 2022)
ZnO	*Mangifera indica L.*	Increased NPK in the leaves and total soluble sugar, carbohydrates, and proline	(Elsheery et al. 2020)
SiO_2_	*Solanum tuberosum*	Reduce the negative impact of water shortage on potato output and improve the growth parameters	(Seleiman et al. 2023)
ZnO	*Solanum melongena*	Increased chlorophyll and relative water content, biomass	(Semida et al. 2021)
ZnO	*Solanum lycopersicum*	Improves tomato growth and antioxidant enzyme activity under stress conditions.	(El-Zohri et al. 2021)
Fe	*Fragaria ananassa*	PEG-induced water stress hurts morphological and physiological features by boosting antioxidant enzyme activity and decreasing MDA and H_2_O_2_	(Yosefi et al. 2022)
Si	*Coriandrum sativum*	Coriander plants provided a rapid and very effective approach for determining content and enzyme activity in both stressed and non-stressed plants	(Afshari et al. 2021)
SiO_2_	*Pisum sativum*	Increase pea growth under drought conditions via activating antioxidant mechanisms, lowering ROS, increasing water content and leaf area, and decreasing root-to-shoot ratio	(Sutulienė et al. 2021)
ZnO	*Zea mays*	Melatonin production and antioxidant enzyme system activity are increased, as is the relative transcript abundance of SOD, CAT, APX, CAT, TDC, and SNAT	(Sun et al. 2020)
TiO_2_	*Triticum aestivum*	Increased germination rate, as well as root and shoot fresh weight and vigor index. Reduced average germination time, increased seedling development	(Faraji & Sepehri 2019)
C	*Capsicum annuum*	Under drought stress, exogenous application enhanced relative water content, chlorophyll-related parameters, proline content, and antioxidant activities	(Alluqmani & Alabdallah, 2023)
Co	*Glycine max*	Relative water content, drought tolerance index, and biomass reduction rate all improved. Upregulation of drought-responsive gene expression, particularly GmERD1, in both roots and shoots	(Linh et al. 2020)
SiO_2_	*Triticum aestivum*	PSII shoot biomass, root biomass, shoot water content, and quantum yield were all reduced	(Potter et al. 2021)
TiO_2_	*Linum usitatissimum*	Avoid cell membrane damage and increased protein content	(Aghdam et al. 2016)
Fe	*Triticum aestivum*	Reduced oxidative stress by switching antioxidative mechanism	(Arikan et al. 2022)
SiO_2_	*Fragaria ananassa*	Enhanced enzymatic activity, root, and shoot biomass	(Zahedi et al. 2020)

### NPs mediated molecular responses to drought stress in plants

NPs influence drought stress responses in plants by regulating specific genes and pathways involved in stress tolerance. The P5CS gene, essential for proline biosynthesis, enhances drought resistance by improving osmotic balance under water deficit conditions (Pérez-Labrada et al., 2020). Similarly, AREB/ABF transcription factors, regulated by abscisic acid, play a pivotal role in activating drought-responsive genes (Yoshida et al., 2015). Downregulation of the ZFHD gene alleviates drought stress by modulating the abscisic acid biosynthesis pathway, while TAS14 downregulation reduces osmotic pressure and increases solute aggregation, including potassium ions and sugars, enhancing drought resilience (Pérez-Labrada et al., 2020). These molecular mechanisms collectively strengthen plant tolerance to drought stress.

### Temperature stress

Rising temperatures are among the most detrimental environmental stressors impacting agricultural plants (Fahad et al., 2017). Heat stress induces oxidative stress, producing ROS; these ROS drive the peroxidation of membrane lipids, disturb cellular homeostasis, and hinder several metabolic processes, finally manifesting as cell death (Fahad et al., 2017; Afzal et al., 2020). Additionally, heat stress adversely affects photosystem II, electron transport, carbon fixation, and chlorophyll stability, all of which are essential for the development and production of plants (Shang et al., 2019b).

The application of SeNPs has shown potential in enhancing the morphological characteristics of wheat under heat stress morphological features of wheat under heat stress (Omar et al., 2023). As nano-fertilizers, metallic NPs can enhance plant resilience by improving nutrient uptake and physiological responses. When applied in varying doses, NPs have been found to promote plant growth and hydration under heat-stress conditions. NPs exhibit antioxidative properties at low concentrations, while high concentrations may lead to oxidative stress (Ali et al., 2021a). Under heat stress, AgNPs dramatically improve the morphological features of wheat plants. Metallic NPs used as nano-fertilizers for sustainable agriculture may help plants withstand heat stress (Arora et al., 2022) (**Table 3**). Provided a detailed summary of NPs and their effects on heat stress.

**Table 3:** Effect of nanoparticles on enhancing plant responses to salinity stress.

NPs	Plants	Effect on plants	References
SiO_2_	*Ocimum basilicum*	Enhanced fresh and dry weight and increased chlorophyll content	(Farouk et al., 2020)
SiO_2_	*Lens culinaris Medik*	Increased seeds germination rate and improve plants growth	(Sarkar et al. 2022)
Cu	*Solanum lycopersicum*	Cu-NPs sprayed on fruits can mitigate the harmful effects of NaCl treatment. This benefit is most significant when the fruits are stored at approximately 24°C	(Hernández-Fuentes et al. 2017)
nSiO_2_	*Fragaria ananassa*	Improves water loss, keeps chlorophyll levels stable, and helps in water retention	(Avestan et al. 2019)
Cs–Se	*Momordica charantia*	Boosting photosynthetic pigments, photosynthesis, growth, and yield	(Sheikhalipour et al., 2021)
Carbon	*Lactuca sativa*	Reduce the negative influence of salt stress on seed germination	(Baz et al. 2020)
ZnO	*Solanum melongena*	Improve growth, chlorophyll contents, total soluble sugars, proteins, and free amino acids.	(Anwar et al., 2023)
Se	*Fragaria ananassa*	Improved antioxidant enzyme activity in strawberries, leading to lower stress-induced lipid peroxidation and H_2_O_2_ levels	(Zahedi et al. 2020)
CeO_2_	*Oryza sativa*	Shoot length, chlorophyll content, and fresh and grain weight are all increased	(Zhang et al., 2021)
Mn	*Vigna radiata*	The indicator of membrane stability, chlorophyll concentration, and nitrate reductase activity all improved	(Etesami et al. 2021)
CeO_2_	*Brassica napus*	Increasing chlorophyll content, carbon absorption, fresh weight, and leaf size	(Li et al. 2022)
TiO_2_	*Solanum lycopersicon*	Inhibition of growth, increase in growth, yield, and quality	(Khan et al., 2017)
Au	*Triticum aestivum*	Improved chlorophyll content, defense system, growth properties	(Wahid et al. 2022)
ZnO	*Vicia faba*	Enhanced the accumulation of photosynthetic pigments, nutrients, amino acids, antioxidants	(Mogazy & Hanafy 2022)
Fe_2_O_3_	*Eucalyptus tereticornis*	Increased growth, biochemical alterations, increased SOD, sugar, and proline levels, improved gene expression of antioxidant enzymes, increased shoot length	(Singh et al., 2021a)
SiO_2_	*Cucumis sativus*	Ion homeostasis, stomatal opening control, increased K+ absorption and K+/Na+ ratio, and improved water and intake of nutrients	(Alsaeedi & Show 2019)
Ag	*Pennisetum glaucum*	Decreased oxidative damage by boosting antioxidant enzyme activity, up-regulating metabolic processes	(Khan et al. 2023)
ZnO	*Vicia faba*	Enhanced the accumulation of photosynthetic pigments, nutrients, amino acids, antioxidants	(Shah et al. 2022)
Fe_2_O_3_	*Helianthus annus*	Improved activities of APX, CAT, and POD enzymes, chlorophyll, photosynthetic rate	(Nawaz et al. 2023)
ZnO	*Brassica napus*	Boosted the amounts of chlorophyll MDA, H_2_O_2_, and proline	(Farouk et al., 2020)
CuO	*Solanum lycopersicon*	Increased amounts of vitamin C, phenols, glutathione, increased antioxidant activity, better Na+/K+ ratio	(Pérez-Labrada et al. 2019)
ZnO	*Gossypium barbadense*	Improve mineral contents and growth rate	(Hussein & Abou-Baker 2018)
ZnO	*Solanum lycopersicum*	Decreased stress, improve growth, photosynthesis and antioxidant enzymes	(Faizan et al. 2021)

### Molecular mechanisms of nanoparticle-mediated heat stress tolerance in plants

Plants respond to heat stress by synthesizing heat shock proteins (HSPs) and molecular chaperones that stabilize proteins under extreme conditions (Rawat et al., 2021). When NPs were applied to plants in various doses to reduce the effects of heat stress, an enhancement in plant growth and hydration was observed. When NPs are delivered to plants in low concentrations, they exhibit antioxidant properties; however, at high concentrations, oxidative stress occurs. Numerous studies have documented that multiwall carbon nanotubes facilitate the production of HSPs, including HSP90, and augment the expression of heat shock genes (Khan et al., 2017; Zhao et al., 2023). Additionally, it has been demonstrated that cerium oxide nanoparticles (CeO_2_NPs) increase HSP70 levels in maize, contributing to improved heat stress tolerance. NPs also mitigate heat stress by regulating stomatal opening, thus enhancing transpiration and cooling (Ali et al., 2021a).

In soybean, ZnO NPs enhanced photosynthetic pigments, proline accumulation, and antioxidant enzyme activity by upregulating stress-related genes like HSF-34 and WRKY1 (Mirakhorli et al., 2021). Copper-based NPs modulate genes involved in oxidative stress, brassinosteroid biosynthesis, and root formation, while also influencing secondary metabolite production for signaling and defense (Ali et al., 2019). Similarly, silicon and lanthanum oxide (La₂O₃) NPs regulate aquaporin gene expression, improving water content and stress tolerance in wheat and maize (Yue et al., 2017; Ali et al., 2019).

### Salinity stress

Global warming has exacerbated water shortages, leading to the increased reliance on saline water for irrigation in agricultural regions, which in turn increases soil salinity. This practice, however, contributes to rising soil salinity levels, posing a substantial challenge in modern agriculture. High salinity impairs plant growth by disrupting water and nutrient uptake, and prolonged exposure can ultimately result in plant mortality (Al-Khayri et al., 2023). When sodium chloride (NaCl) concentrations exceed 200 mM, salinity affects all stages of a plant’s life cycle, such as seed development, seedling growth, vegetative growth, and flowering (Yuan et al., 2019).

Salinity is a critical abiotic stressor that limits food production and adversely affects the quality of crops, hindering their ongoing development. It remains a major obstacle to achieving sustainable agricultural production, as recognized by the scientific community. Salinity stress affects 20% of all cultivated land globally, and the proportion is increasing on a daily basis (Kumar et al., 2022). The great majority of agricultural plant species are glycophytes, which are especially subject to salt stress and, hence, constitute the most significant environmental abiotic stress that may significantly affect crop yield (Munns and Tester, 2008). The majority of salinity issues are excessive sodium chloride, widely found in soils and water supplies in coastal and arid regions (Hailu and Mehari, 2021). Elevated NaCl concentrations present several challenges for higher plants. These include (i) it increases osmotic pressure in the external solution, necessitating osmotic adjustment by plant cells to prevent dehydration; (ii) Potential interference of excess sodium with the absorption and transportation of vital ions like potassium (K^+^) and calcium (Ca^+2^); and (iii) causing direct toxic effects on cell membranes and enzymes due to the presence of sodium (Na^+^) and chloride (Cl^-^) (Knop et al., 2023).

Salinity stress diminishes the soil’s osmotic potential, disrupts the nutritional equilibrium, and elevates ionic toxicity (Truscă et al., 2023). NPs play a significant role in mitigating salt stress in plants. They help regulate ion balance, reduce sodium ion toxicity, and enhance potassium ion absorption. Additionally, NPs activate antioxidant defense systems and improve pigment composition, solute levels, and stomatal conductance. For instance, magnetite NPs increase chlorophyll content and antioxidative enzyme activity, contributing to salinity resistance in wheat (Rawat et al., 2021). Similarly, nano-silicon dioxide (SiO_2_) has been demonstrated to enhance growth and antioxidant activities in Glycine max and improve seed germination and growth in wheat cultivars under salt stress (Dhakate et al., 2022).

ZnO NPs were applied to salt-stressed *Brassica napus* plants to mitigate the harmful effects of salinity by enhancing the antioxidative system, promoting osmolyte production, and regulating ionic balance (Zulfiqar and Ashraf, 2021b). Cu NPs foliage sprayed on *Solanum lycopersicum* boosted growth while reducing the impact of salt stress. Cu NPs increased levels of glutathione, polyphenols, and vitamin C, and altered the activity of APX, GPX, and SOD, contributing to improved overall plant growth and development (Fu et al., 2023). Seed priming with ZnO NPs minimized the adverse effects of NaCl treatment on Lupinus termss by increasing pigment levels, modulating osmoregulation, and lowering stress-related metabolite concentrations. According to another report, seed priming *T. aestivum* L. with AgNPs reduced salt stress (Hossain et al., 2021) (**Table 4**). represents the role of NPs in mitigating salinity stress.

**Table 4:** Effect of Nanoparticles on Enhancing Plant Responses to Temperature Stress.

NPs	Plants	Effect on plants	References
Se	*Lycopersicum esculentum*	Enhanced morphological growth characteristics	(Ishtiaq et al. 2023)
Se	*Sorghum bicolor*	Increased thylakoid and photosynthetic apparatus integrity	(Haghighi et al. 2014)
TiO_2_	*Cicer arietinum*	MDA levels and the electrolyte leakage index were reduced	(Noohpisheh et al. 2020)
Ag	*Triticum aestivum*	The genes responsible for antioxidant activity were upregulated	(Azimi et al. 2014)
Si	*Agropyron elongatum*	Seed dormancy was reduced, seed germination was raised, and seedling weight was increased	(Khan et al., 2017)
ZnO	*Oryza Sativa*	Plant growth was stimulated, oxidative stress was minimized, and antioxidative system gene expression was increased	(Song et al. 2021)
TiO_2_	*Cicer arietinum*	Lessened membrane damage indexes, improved redox status	(Ghabel & Karamian 2020)
Zn	*Triticum aestivum*	Increased antioxidant enzyme activity and yield	(Hassan et al. 2018)
ZnO	*Triticum aestivum*	Biomass, photosynthetic pigments, soluble sugars, protein, and indole acetic acid levels have all increased	(Azmat et al. 2022)
Ag	*Triticum aestivum*	Enhanced plant morphological characteristics	(Iqbal et al. 2020)
TiO_2_	*Cicer arietinum*	Reduced H_2_O_2_ content, Photosynthetic activity increased	(Hasanpour et al. 2015)
ZnO	*Oryza Sativa*	Decrease oxidative stress, improve antioxidative system's gene expression	(Song et al. 2021)
Fe	*Triticum aestivum*	Reduced oxidative stress and increased production and activity of antioxidant enzymes	(Naeem et al. 2022)

### Molecular modifications in plants mediated by NPs under salinity stress

**Figure 4:**
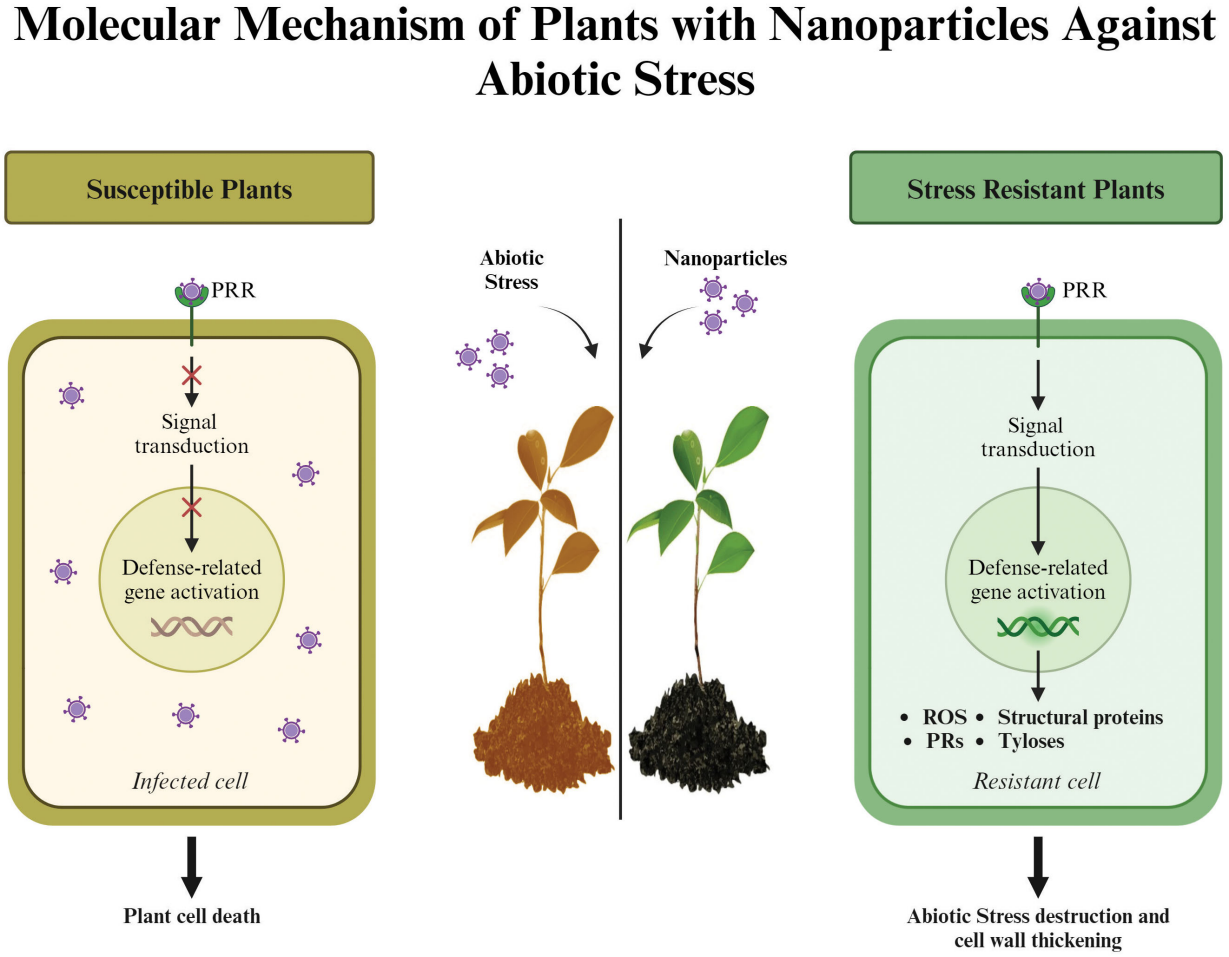
Different types of abiotic stresses like temperature, drought, heavy metals, salinity and flooding effects on plants and their response in various ways.

Molecular processes in plants play a critical role in determining their biological functions, particularly under stress conditions (**Figure 4**). The impact of NPs on these processes, including gene expression and cellular activities, is evident and essential for their effectiveness. For example, salinity stress alters gene expression, which in turn affects plant growth and cellular functions. In NP-mediated root development, reduced miR164 expression influences auxin hormone signaling, while increased miR169 and decreased miR167 expression promote lateral root formation and accelerate flowering (Tolaymat et al., 2017). Foliar application of Zn NPs in rapeseed (*Brassica napus* L.) under salinity stress downregulated stress-related genes such as SKRD2, MYC, and MPK4, while upregulating ARP and MPK, which regulate hormonal and physiological responses (Hezaveh et al., 2019). Similarly, silicon nanoparticles (Si NPs) enhanced growth and molecular adaptations in *Cannabis sativa* L. under salinity stress (Guerriero et al., 2021). In tomato plants, proteomic studies revealed that Si NPs affected genes involved in light-harvesting complexes, cytochrome b6f (Cytb6f), and ATP synthesis (Guerriero et al., 2021).

### Role of NPs in modulating HM transport and stress alleviation in plants

Agriculture faces persistent issues with the buildup of HMs in the soil as a result of increasing industrialization, notably in mining and tanning industries (Sarwar et al., 2017). HM in soil is a contemporary issue since it is difficult to dissolve, readily translatable, and extremely hazardous to public health and the environment. HMs changes the natural composition of the soil (Yan et al., 2020; Thakare et al., 2021). The main contaminants that negatively impacting plant’s agro-biological systems are Cr, Cd, Ni, HG, Pb, and Cu (Yaashikaa et al., 2022). Due to their oxidative states, HM are very reactive and induce molecular and cellular alterations, such as changes in plant physiology with enzyme deactivation and protein denaturation, as well as substituting essential metals and damaging membranes (Ghori et al., 2019). These changes reduce photosynthesis and affect plant enzyme activity.

HM-induced oxidative stress causes plant HM resistance due to the high reactivity of HM, which drives at both molecular and cellular levels. HMs can disrupt plant physiology by inactivating enzymes, denaturing proteins, substituting essential metals, and damaging cellular membranes (Ghori et al., 2019). These disruptions lead to reduced photosynthesis and impaired enzyme activity, ultimately affecting plant growth and survival.

NPs present a great potential to improve plant tolerance to HM stress. NPs can be supplied to plants via foliar sprays or soil amendments. For example, selenium and silicon nanoparticles have been shown to alleviate Cd and lead (Pb) stress in Oryza sativa L. by reducing metal uptake and toxicity (Ahmad et al., 2022). By adding to being treated through the soil, NPs can also be applied to plants through the foliage. For example, selenium and silicon nanoparticles applied to leaves have been shown to reduce Cd and Pb stress in *Oryza sativa* L (Ahmad et al., 2023).

Plants have evolved mechanisms to maintain homeostasis in managing metal absorption, detoxification, and transport. NPs enhance these mechanisms by lowering the bioavailability of metal pollutants, influencing the regulation of genes responsible for metal transport, and strengthening the apoplastic barrier that prevents metals from entering the roots. The binding of NPs to HMs in plant cell walls primarily occurs through adsorption and complexation processes. Plant cell walls contain a variety of functional groups, including hydroxyl, carboxyl, and amino groups, which have a high affinity for binding metal ions (Zhao et al., 2012). When NPs are introduced, they interact with these functional groups and increase the cell wall’s capacity to immobilize HMs through ionic and covalent bonds (Ma et al., 2019). This binding reduces the bioavailability and mobility of metals within plant tissues, effectively sequestering them in a non-bioactive form (Wang et al., 2021). NPs can form stable complexes with metals like Cd, which minimizes their transport across cellular membranes and thus reduces their toxicity. This stabilization is crucial for the plant’s detoxification mechanisms, as it limits the potential of HMs to disrupt cellular functions (Li et al., 2023). By enhancing these metal-binding interactions within the cell wall matrix, NPs support the plant’s natural defense mechanisms against metal stress (Khan et al., 2021).

Although it does not completely block contaminants, the apoplastic barrier performs crucial protective activities in plant roots by managing the passage of ions, oxygen, and water (Fiol et al., 2021). The apoplastic barriers prevent HMs from entering plant roots, and their effectiveness can be increased by NPs (Singh et al., 2021a). By forming complexes with the HMs in the cell walls, NPs bind to them and render them inactive. Studying the many aspects of HM stress relief requires interaction between NPs and HMs. The application of NPs has been supported due to the decreased mobility and resulting bioavailability of metal pollutants in the soil. For instance, adding Si NPs and Fe_3_O_4_ NPs makes Cd more stable and reduces its mobility (Rahman et al., 2022). When NPs complexes are adsorbed, they become stationary and inhibit the movement of HMs inside plants, which lowers the biological activity of the plants (Alghanem et al., 2022). NPs improved the biosynthesis of these protective organic acids, as it was demonstrated when Si NPs were utilized to lessen the harm brought on by Cd (Mukarram et al., 2022). Additionally, NPs enhance the properties of soil; for instance, hydroxyapatite NPs can increase soil pH and releases phosphate, which lowers HM toxicity. NPs with a high surface-to-volume ratio, NPs can interact with specific cellular biomolecules and activate several biochemical pathways (Nel et al., 2009).

Similar outcomes were shown when Cd was remedied in coriander using nano-TiO_2_. These findings included decreased Cd concentration, decreased oxidative injuries brought on by Cd stress, and enhanced agronomic features. In soybeans, the application of nano-TiO_2_ enhanced photosynthetic rate and growth metrics. The use of Si NPs resulted in an increase in biomass due to a reduction in Cd stress (Memari-Tabrizi et al., 2021). To mitigate the effects of Cd on rice, graphite carbon nitride was generated; as a consequence, there was a large rise in plant biomass and a major decrease in Cd-induced toxicity (Hao et al., 2021).

NPs influence HM transport in plants through species-specific mechanisms that regulate stress responses. For example, silicon nanoparticles (Si NPs) reduce Cd toxicity in rice by downregulating Cd uptake and transport genes, such as low-affinity cation transporter (LCT1) and natural resistance-associated macrophage protein 5 (NRAMP5), while upregulating genes like HM ATPase 3 (HMA3) for Cd sequestration in vacuoles (Cong et al., 2019). Si NPs also enhance silicon uptake via the LSI1 gene, further mitigating Cd accumulation. Similarly, iron oxide (FeO) and hydrogel NPs reduce the expression of key Cd transporters, including OsHMA2, OsHMA3, and OsLCT1, lowering Cd translocation in rice (Ahmed et al., 2021). The NRAMP gene family, responsible for HM transport across species, is also influenced by NPs. Nanoscale zero-valent iron (nZVI) has been shown to alleviate HM buildup by downregulating metal uptake genes (e.g., IRT1, IRT2, YSL2, YSL15), promoting plant growth and reducing metal stress (Guha et al., 2020). (**Table 5**) summarizes the influence of NPs on alleviating HM stress.

**Table 5:** Role of nanoparticles in alleviating heavy metal stress in plants.

NPs	Plants	Effect on plants	References
ZnO	*Oryza sativa*	Lower level of Cd and as in roots and leaves	(Ma et al. 2020)
Si	*Pisum sativum*	Cr phytotoxicity and oxidative stress were reduced	(Tripathi et al. 2015)
Fe_2_O_3_	*Vigna radiata*	Lowered As uptake and toxicity, improve chlorophyll content	(Shabnam et al. 2019)
TiO_2_	*Coriandrum sativum L.*	Reduced oxidative stress, ROS scavengers	(Sardar et al. 2022)
Si	*Satureja hortensis L.*	Reduce Cd level and increase plant growth	(Memari-Tabrizi et al., 2021)
Cu	*Brassica*	Enhanced photosynthetic rate, antioxidant enzymes activity	(Wang et al. 2022)
ZnO	*Abelmoschus esculentus*	Chlorophyll, proline, and antioxidant enzyme levels rise	(Rajpal et al., 2023)
CeO_2_	*Oryza sativa*	Shoot length, chlorophyll content, and fresh and grain weight are all increased	(Zhou et al. 2020)
Ag	*Lilium*	Increased leaf and bulb biomass, improved development, and blooming Chlorophyll content in leaves has increased	(Salachna et al. 2019)
Fe_2_O_3_	*Dracocephalum moldavica*	Increase in root and shoot fresh and dry weight, leaf length and leaf area, increased amino acids and enzyme activity	(Moradbeygi et al. 2020)
TiO_2_	*Phaseolus vulgaris*	Mycorrhizal colonization in root tissues, arbuscular frequency, and AMF relative density all increased	(El-Gazzar et al. 2020)
MgO	*Daucus carota*	Detoxify ROS to reduce lead (Pb) stress and boost plant development through increased antioxidant enzyme activity	(Faiz et al. 2021)
Si	*Coriandrum sativum*	Reduced the harmful effects of lead (Pb) on coriander plants by lowering Pb concentrations and strengthening the plant's defensive mechanism.	(Fatemi et al. 2021)
Si	*Triticum aestivum*	Enhanced plant growth, photosynthesis, and SOD and POD activity; decreased H_2_O_2_, EL, MDA, and Cd content	(Thind et al. 2021)
SiO_2_	*Glycine max*	Increased chlorophyll concentration; decreased Hg deposits in roots	(Li et al., 2021b)
Fe_2_O_3_	*Oryza sativa*	Increased plants height; improved fresh and dry biomass	(Ahmed et al., 2021)
Cu	*Triticum aestivum*	Decreased Cr availability, increased nutritional absorption, antioxidant content	(Noman et al. 2020)
ZnO	*Oryza sativa*	ZnO treatment boosted seedling growth	(Yan et al. 2021)
TiO_2_	*Zea mays*	Cd buildup is reduced, and antioxidant enzyme activity is increased	(Lian et al. 2020)
MgO	*Zea mays*	Reduced HM toxicity and increased plant growth	(Fouda et al. 2021)
FeO	*Oryza sativa*	Reduced As deposits, Fe uptake increased, and photosynthetic pigments were restored	(Bidi et al. 2021)
TiO_2_	*Coriandrum sativum*	Reduced oxidative damage and increased proline and yield biosynthesis	(Sardar et al. 2022)
TiO_2_	*Glycine max*	Improve the root, shoots biomass and boost yield	(Hussain et al. 2021)
FeO	*Triticum aestivum*	Cd toxicity was reduced, and growth, yield, and chlorophyll content were all enhanced	(Manzoor et al. 2021)

### Environmental risks of nanoparticles in agriculture: coping with abiotic stress

The potential for nanotechnology is to boost agricultural production and output, protect crops from environmental challenges, and lessen the release of chemicals into the environment. The distinct effects of nanoparticles (NPs) are significantly impacted by various factors, including their genotype or cultivar, size, dosage, composition, surface area, surface coatings, redox state, and the methods used for their application. However, some plant species, such as soybean, sorghum, broad bean, sweet basil, tomato, wheat, onion, and barley, have shown toxicity to NPs like CeO_2_, Se, TiO_2_, and ZnO (Vasseghian et al., 2022).

An increasing amount of research indicates that NPs could potentially impact higher-level consumers, including humans. Recent studies are exploring not only the toxicity of NPs to plants but also their wider implications (Rajpal et al., 2023). *E171* (TiO_2_ NPs-based food additive) has recently been demonstrated to enhance gastrointestinal tumor development and progression in a mouse model by altering systems such as inflammation, immunological responses, cancer signaling, and cell cycle (Li et al., 2021a). Plants suffer a range of adverse effects from NPs, such as, reduced seed germination and root elongation, biomass, growth inhibition, limited mineral uptake, ROS, generation of electron-hole pairs, damage to the cell wall and cell membrane, impairment of cellular structures, and NP aggregation, which leads to increased ROS levels and tissue toxicity (Ghori et al., 2019). However, observations showed that the toxicity of NPs is conditional on things like the plant species and genotype involved, the NPs’ concentration and size, and the length of time the plants are exposed to them (Zhou et al., 2023b).

**Figure 5:**
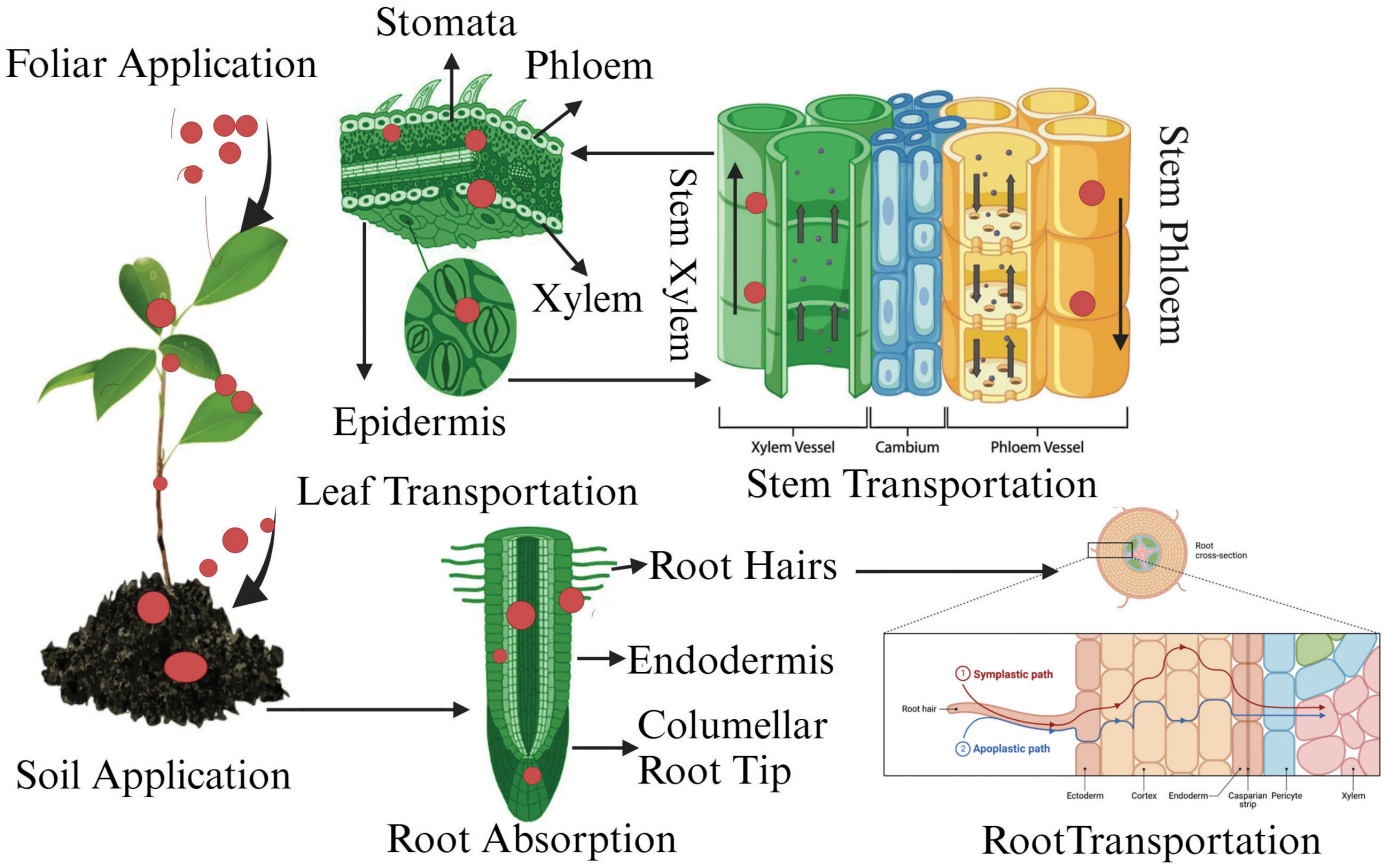
Pathways of nanoparticle uptake, transport, and distribution in plants: from root absorption to foliar application.

Researchers in medicine and ecology have gained substantial insights into the protein corona, but its role in plants has only recently been clarified. The absorption, translocation, effects, and ultimate fate of NPs in plants are influenced by various factors, including the protein corona that surrounds them (Huang et al., 2017). The protein corona refers to a dynamic layer of proteins and other biomolecules that adsorb onto the surface of NPs when they enter biological environments, including plant systems. The formation of the protein corona is critical because it alters the physicochemical properties of NPs, such as their size, charge, and surface characteristics, which can impact how they are recognized and transported within plant tissues (Wang et al., 2020). In particular, the protein corona affects the uptake, translocation, and bioavailability of NPs in plants by modulating their interaction with cell walls and membranes (**Figure 5**). For instance, certain proteins in the corona may facilitate or inhibit NP entry into cells by interacting with membrane receptors or transporters, impacting the NP’s eventual localization within plant tissues (Kumar et al., 2022). Additionally, the protein corona can influence the biocompatibility and potential toxicity of NPs in plants, as specific proteins may reduce or increase the plant’s stress response to foreign particles (Petersen et al., 2016). Thus, understanding the formation and composition of the protein corona in plant systems provides valuable insights into the behavior and fate of NPs in agricultural and environmental contexts. This knowledge helps to better predict how NPs interact with plants at the cellular and molecular levels, which is essential for assessing their risks and benefits in agricultural applications.

Before nanobiotechnology can be commercially applied, thorough research is essential, as cells respond differently to various aspects of the technology, such as its introduction, uptake, translocation, accumulation, concentration, surface activity, variability, and most importantly, the association of NPs with cellular organelles and their impact on cellular metabolism (Ali et al., 2021a). Given these limitations, future research efforts should investigate ways to make agricultural NP usage safer and more productive. To reduce the potential for ecotoxicological risks to humans and plants, greater research into nano-bio interaction and impact evaluations is needed. The safe disposal of NPs is another critical issue that requires the focus of the scientific community. If not burned, NPs should be disposed of in sealed containers in a specially designated place for hazardous waste. Unfortunately, NPs are being employed at several labs/institutions worldwide that do not have access to suitable technology. The usage and disposal of NPs require rigorous safety standards and regulations. The current evaluation stresses the need for precautionary measures while using NPs in the present setting (Donia and Carbone, 2019).

### Future challenges

**Figure 6:**
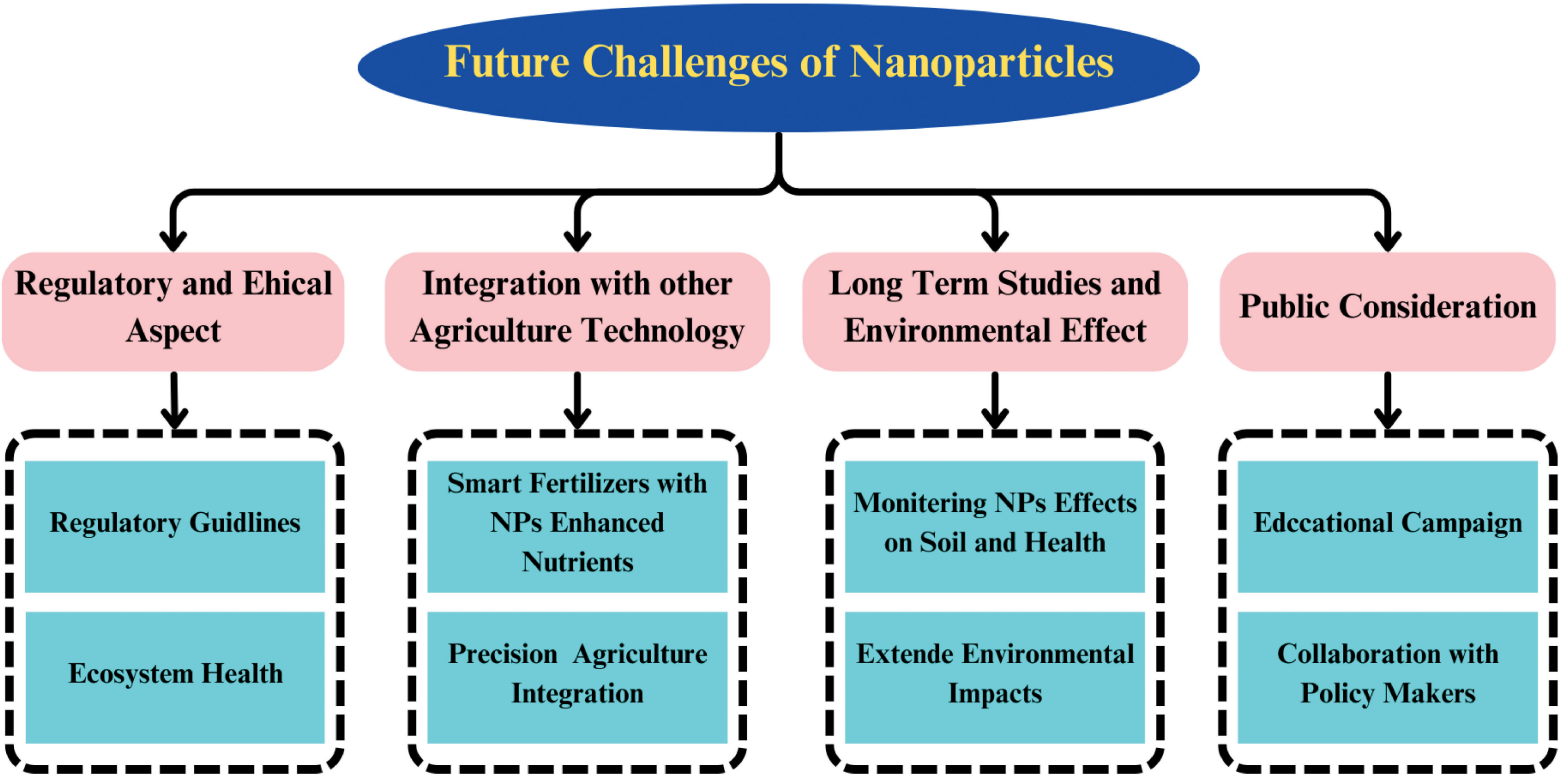
A schematic diagram illustrate the uptake and translocation mechanism of Nps in plants.

Environmental degradation poses a major risk to global food security, influenced by issues such as soil erosion, nutrient loss, pesticide pollution, and diminishing biodiversity. These obstacles, combined with the continuous reliance on conventional agricultural techniques, highlight the need for innovative approaches. Nanotechnology emerges as a viable solution, capable of bolstering crop resilience, improving nutrient distribution, and alleviating the effects of environmental challenges, thus supporting sustainable farming practices.in addition, abiotic stressors such as drought, salinity, and temperature extremes are becoming more frequent and severe. These stressors significantly reduce crop yield and compromise agricultural productivity. NPs have emerged as promising tools to alleviate plant abiotic stress by enhancing nutrient mobility, providing protection against damage, and improving stress tolerance. NPs are a promising new technology for improving agricultural sustainability. However, there are still some challenges that need to be addressed before NPs are widely adopted (**Figure 6**). These challenges include:

### Regulatory and ethical aspects

Nanotechnology’s integration into agriculture, an emerging field, requires thorough attention to both regulatory and ethical concerns. Developing well-defined guidelines and regulations is essential to guarantee the safe application of NPs in agricultural systems. These measures are crucial for minimizing possible environmental and health risks related to NP applications. Ethical considerations include the impact of large-scale NP use on food safety, biodiversity, and ecosystem health. Collaboration among governments, regulatory bodies, and researchers is vital to determine safe NP concentration thresholds for various agricultural applications.

### Integration with other agricultural technologies

Nanotechnology can be integrated with other emerging agricultural technologies, such as Precision Agriculture, to enhance efficiency. Technologies like drones, sensors, and satellite imagery facilitate targeted nutrient and pesticide delivery. However, there is ongoing debate about the environmental impact and efficiency of these technologies, partly due to NP concentrations. Precision Agriculture could benefit from NP-enhanced nutrients, allowing for more efficient and less polluting delivery systems. Additionally, smart fertilizers using NPs can provide variable delivery routes and amounts based on plant needs, promoting sustainability.

Integrating nanotechnology with other advanced agricultural technologies, particularly within the framework of Precision Agriculture, holds significant potential for improving efficiency and sustainability in farming. Precision Agriculture technologies, such as drones, soil sensors, and satellite imagery, enable real-time monitoring of crop health, soil quality, and environmental conditions. These tools facilitate targeted applications of fertilizers and pesticides, reducing waste and limiting environmental impact (Getahun et al., 2024). NPs could further enhance these technologies by improving the formulation of agrochemicals, enabling controlled-release fertilizers, and offering nano-based pesticides that are more precise and require lower doses than traditional formulations (Zain et al., 2023).

One promising application is the use of NP-enhanced nutrients, which could allow for more efficient delivery systems by controlling the release of nutrients directly into the root zones, minimizing runoff and reducing groundwater contamination (Shanmugavel et al., 2023). This approach aligns with sustainable practices by delivering nutrients in a manner that minimizes pollution. Additionally, integrating NPs with smart fertilizers that respond to environmental cues or plant needs could provide tailored nutrient delivery. For instance, NPs can be engineered to release nutrients in response to soil moisture levels or pH changes, thus ensuring that plants receive the right nutrients at the right time (Wang et al., 2020).

However, concerns regarding the long-term environmental and ecological impact of NPs persist, especially with respect to NP accumulation in soil and water systems. Research has indicated that while nanomaterials offer efficiency, their interactions with various environmental factors are complex and not fully understood, raising questions about their fate and effects in ecosystems (Lead et al., 2018). Ongoing studies aim to address these uncertainties by examining the biodegradability, persistence, and toxicity of NPs used in agricultural settings, aiming to establish guidelines for safe concentrations and applications.

Through strategic integration, nanotechnology could enhance the effectiveness of Precision Agriculture by ensuring that nutrients and pesticides are applied only where and when needed, promoting sustainable agricultural practices. This synergy between nanotechnology and digital farming tools could ultimately lead to a more environmentally conscious approach to food production, potentially mitigating some of the environmental pressures posed by conventional agricultural methods (Zhang et al., 2021).

### Advance methodologies to track and observe the NPs delivery in plants

Advanced methodologies for tracking and observing NPs delivery within plants have significantly progressed, enabling detailed insights into NPs uptake, distribution, and transformation. Techniques such as confocal microscopy with intrinsically fluorescent or dye-labeled NPs (Demirer et al., 2020; Santana et al., 2020), provide real-time tracking of NPs, allowing visualization of their transport across cellular barriers and into specific plant structures, including roots and leaves (Santana et al., 2020; Jeon et al., 2024). Another powerful approach involves laser ablation coupled with inductively coupled plasma mass spectrometry (ICP-MS), which can quantitatively analyze elemental composition and map the distribution of metal-based NPs within different plant tissues, providing spatial resolution of NP presence in roots, stems, and leaves (Avellan et al., 2019).

Synchrotron-based X-ray fluorescence (XRF) imaging (Stegemeier et al., 2017) and X-ray absorption spectroscopy (López-Moreno et al., 2010) offer high-resolution elemental mapping, enabling researchers to observe the exact location and chemical state of NPs within plant cells and organelles. These techniques are especially useful for assessing NP translocation and transformations at cellular and subcellular levels (Lombi et al., 2016). For three-dimensional analysis, methods like X-ray tomography, magnetic resonance imaging (MRI), and advanced confocal microscopy allow for 3D reconstructions, which facilitate a comprehensive understanding of NP penetration pathways and localization within plant tissues (Staedler et al., 2013; Santana et al., 2020).

Additional techniques such as Fourier-transform infrared (FTIR) spectroscopy, Raman spectroscopy, and microparticle-induced X-ray emission (µ-Pixe) are essential for examining chemical changes in NP surfaces (Larue et al., 2014). These tools help to monitor how NPs interact with plant biomolecules, thus providing insight into the stability and transformations of NPs once inside the plant environment (Larue et al., 2014). Furthermore, single-particle ICP-MS is used to characterize individual NPs within plant tissues, offering data on size, distribution, and potential aggregation, which are critical for understanding NP behavior after uptake (Keller et al., 2018). Collectively, these advanced methodologies allow researchers to accurately track, quantify, and analyze NPs within plants, which is essential for optimizing nano-enabled delivery systems in agricultural applications.

These innovative tracking tools thus support a detailed understanding of NP delivery, behavior, and impact within plant systems, addressing essential concerns for the development of sustainable and effective NP applications in agriculture.

### Public considerations

Public perception of NPs in agriculture is an important consideration. Concerns often stem from a deficiency of information and fears around the ingestion of NPs in food production. Educational campaigns and collaboration among researchers, policymakers, and the public are essential to foster informed and acceptable NP use in agriculture.

### Long-term studies and environmental effects

Long term studies are crucial for understanding how NPs persist, move, and affect ecosystems within agricultural systems. While short-term studies provide insight into initial effects, only extended-duration research can reveal how NPs behave over time in complex agricultural systems and ecosystems. These studies are especially valuable for organic and sustainable farming systems, where minimal synthetic inputs are emphasized. In such contexts, the introduction of nanoparticles could have unexpected consequences for soil health, water quality, and crop quality.

Long-term field studies examining the environmental impacts of nanoparticles (NPs) indicate that NPs undergo complex transformations influenced by soil chemistry, redox potential, and interactions with plant and microbial metabolites, leading to significant environmental implications. For instance, metal-based NPs such as copper oxide (CuO) can be reduced in acidic, low-oxygen environments, forming stable compounds that persist in soil, thereby altering soil chemistry and affecting nutrient bioavailability over time (Shang et al., 2019a; Arshad et al., 2021). Additionally, transformations within plant systems vary with environmental factors like ionic strength and pH, impacting NP dissolution rates and, consequently, nutrient cycling in the surrounding soil and plant ecosystem (Lv et al., 2019; Zhang et al., 2020).

Studies reveal that these transformations affect the entire plant life cycle, with effects extending across generations. For example, nanoparticles such as silver (Ag) and cerium oxide (CeO₂) have been shown to enhance stress responses in plants, which can be inherited by subsequent generations (Zhao et al., 2022; Zhou et al., 2023a). However, the potential for bioaccumulation and biomagnification of NPs in food chains remains a significant concern, as these particles can migrate from soil to crops and subsequently into higher trophic levels, posing ecosystem health risks (Werlin et al., 2011; Wu et al., 2023).

Long-term environmental exposure studies in realistic soil systems underscore the complex interactions of NPs with soil microbiomes, groundwater, and higher organisms, emphasizing the need for sustainable NP application practices. For example, NPs can aggregate or bind with soil components, forming eco-coronas that mitigate toxicity, but complicate environmental fate and impact based on NP composition and environmental context (Kurepa et al., 2020; Borgatta et al., 2021). Understanding these transformations in agriculareral systems is thus crucial for safe and sustainable nano-enabled agricultural technologies.

### Outstanding questions

1. Which plant developmental stages and species should be prioritized for nanoparticle research to better understand the mechanisms driving plant responses to abiotic stress? How do these responses change across different growth phases?
2. How can we improve the incorporation of nanoparticle methods to get a deeper awareness of the growth processes that control plant responses to severe abiotic stress conditions, hence improving the development of stress-resistant plants?
3. How can we develop advanced climate simulation systems that accurately reflect the impact of nanoparticles on crops in field conditions?
4. What are the environmental and safety risks of nanotechnology, and how may they reduced in order to accelerate agricultural development?
5. What are the long-term effects on stressed plants in changing climates, especially in practical farming environments, and how can these outcomes be predicted and managed?
6. How can we promote nanotechnology cooperation among plant scientists, geneticists, data experts, and agronomists to tackle the challenges involved in cultivating stress-resistant plants using nanotechnology?
7. What policies and strategies can be introduced at both national and international levels to encourage the adoption of nanotechnology in agriculture and contribute to achieving global food security goals, such as the ‘zero hunger’ initiative by the Food and Agriculture Organization (FAO)?

## Conclusion

Scientists must design and implement sustainable solutions to minimize agricultural production losses due to abiotic stress. Nanotechnology is a novel and effective way to raise agricultural productivity and quality and manage global food demand. Several NPs are being researched for their potential role in minimizing abiotic stress-induced loss and enhancing plant development and agricultural output. NPs mitigate abiotic stress by triggering plant defense systems such as ROS production and phytotoxicity. NPs easily permeate plant tissues due to their tiny size, impacting plant morphological, physiological, and biochemical processes, enhancing plant development, and boosting crop output in plants exposed to various abiotic challenges.

Further research is necessary to maximize the benefits of NPs in agriculture and mitigate their potential negative impacts; key areas for investigation include: It is crucial to understand how NPs interact with plants at molecular and cellular stages. This includes studying their effects on metabolic processes and optimizing NPs size and concentration for effective field application. Research should focus on the potential toxic effects of NPs across different plant species. Understanding these effects is essential for developing safe application protocols. Exploring how NPs influence gene expression can provide insights into their role in enhancing plant stress tolerance and resilience.

While NPs offer promising solutions for enhancing agricultural sustainability and resilience, their implementation must be approached with caution. By addressing regulatory, ethical, and environmental considerations, nanotechnology can be safely and effectively integrated into agricultural practices, contributing to global food security and environmental health.
